# Contributions of residents from multiple specializations in managing the COVID-19 pandemic in the largest public hospital Brazil

**DOI:** 10.6061/clinics/2020/e2229

**Published:** 2020-08-14

**Authors:** Fabíola Vieira Duarte Baptista, Marilia Ribeiro de Azevedo Aguiar, Joanne Alves Moreira, Felipe Carvalho Barros Sousa, Glauco Cabral Marinho Plenns, Raif Restivo Simao, Vitor Maia Teles Ruffini, Chin An Lin, Maria do Patrocínio Tenório Nunes

**Affiliations:** Programa de Residencia em Medicina Interna, Hospital das Clinicas HCFMUSP, Faculdade de Medicina, Universidade de Sao Paulo, São Paulo, SP, BR.

## INTRODUCTION

History shows that residents have played different parts in previous epidemics, from the AIDS outbreak to the Ebola outbreak in 2014 (1,2). During the first SARS-COV outbreak in 2003, universities and training programs responded to the health threats imposed by the virus by changing clinical responsibilities, performing educational activities, and allocating residents to services considered to be in need (3).

The novel coronavirus is rapidly spreading and the demands to expand and free-up the capacity for critical care beds, both general and acute, in health services has increased (4-5). This has caused changes to the routine of major hospitals in Brazil, including the largest hospital complex in South America, Hospital das Clínicas of the School of Medicine, University of São Paulo (HCFMUSP). HCFMUSP is one of the main centers treating COVID-19 patients in São Paulo City, the epicenter of the disease in the country (6). It also has the largest number of residents in the country (over 1,600), who serve as an important part of the Hospital’s workforce.

This paper aims to describe the experience of organizing almost 500 residents from 40 different residency programs who were summoned to work in the frontlines of the COVID-19 pandemic in a quaternary hospital in Brazil, and its implications so far on medical residency programs.

### Preparing the Hospital for the Pandemic

In early March, the main building in HCFMUSP which contained 900 beds was directed to the exclusive care of patients suspected or diagnosed with COVID-19. To ensure proper patient care and adequate conditions for medical residents working in the “COVID area,” a team of chief residents from the Internal Medicine Residency Program was summoned to organize the resident task force.

### Summoning residents

The supervisors and chief residents (S/CR) of each residency program were asked to provide volunteer residents to work in the “COVID area.” The residents and their respective S/CR filled a questionnaire about their health, medical skills and aptitudes. The objectives were to create multispecialty residents who would have a low workload (maximum of 48 hours weekly, done in 12 hour shifts with 36 hours rest) and the optimization of personal protective equipment (PPE). Every resident was trained in donning and doffing PPE and participated in an orotracheal intubation workshop. They were divided into teams with working physicians assigned as supervisors then deployed to work in ICUs, wards, and in the emergency department.

Each team had members from multiple specialties, and this was thought to ensure that different people would be able to contribute and aggregate different types of competencies and knowledge for patient care. The Multispecialty Teams are presented in the Appendix Supplementary File.

### Challenges

The following are some of the ten principles set by the American Academy of Family Physicians (AAFP) to ensure optimal safety and wellness for medical learners: clinical services provided on a voluntary basis; assurance of PPE provision and training on how to use it; presence of supervision; including medical learners in decision-making; continued education (for example, using online platforms to maintain weekly journal clubs or case study-based groups); and quality of care in which patient safety and high-quality care must be maintained (7).

One problem was dealing with the animosity and barriers which came up from a great number of supervisors and residents from different subspecialties who were worried about their specialized training. Since most elective surgeries and procedures were suspended, surgical residencies were by far the most impaired. The post-pandemic scenario will most likely result in the need to readapt the length and core skills of medical residency programs.

### Internal Medicine Residents’ point of view

The Internal Medicine Program of HCFMUSP conducts a routine follow up of residents through conducting individual interviews with their 132 current residents (8). The strategy was resumed after major changes in routines had been established, providing a safe and proper space for residents to speak up while trying to minimize burnout. It usually serves as mere demonstration of support and care while letting residents know that the hospital notices them beyond their roles as physicians. However, in this context it was important to highlight that residents:

- showed quick growth in professionalism;
- despite being physically and mentally tired, feel happy and proud about being part of this historical moment;
- realized the importance of their role in patient-care (95% of residents);
- recognized how essential internists are to the health system, especially with regard to the COVID-19 pandemic;
- are anxious about the changes and possible deficits in their professional training;
- are preoccupied about the reduced theoretical content during the pandemic;
- fear becoming ill; and
- miss their families, friends, and previous lives.

### Lessons learned

Healthcare workers have a duty to care for the sick. By freely deciding to enter medicine, doctors have implicitly agreed to accept the risks (9). In a pandemic, physicians must be protected when they are called upon to practice outside of their area of expertise or jurisdiction.

Most patients had moderate to severe symptoms of colds as well as other advanced chronic diseases (COVID-19 risk factors), therefore residents whose senior staff were less experienced or motivated in these types of conditions were usually more susceptible to complaints.

When guided under proper supervision and medical training, and if safety measures are ensured, residents may improve their professionalism and altruism in the healthcare field. The opposite is also true, if they are exposed to unprofessional educators.

Competencies developed during the pandemic include: the identification of a potential health threat and risk characterization; epidemiologic investigation; environmental monitoring; laboratory analysis; policy development, adaptation, and implementation; organization of medical service; clinical and communication skills; and most importantly, bioethical experience by providing excellent care following the principle of beneficence.

Therefore, it is evident that the competencies acquired so far are unique and that residents will experience uncertainties in treating patients in social isolation. This is something that will probably mold their professional identity and influence their career choices. Furthermore, most residents demonstrated the fear of getting the disease and of passing this on to their families, as well as feelings of anxiety and vulnerability (10). Other important concerns included their fear of losing too much time for training for their specialization and possible delays in their residency programs.

## CONCLUSION

Our experience shows that so far, residents from different medical programs are capable and ready to work in a challenging environment such as that of the COVID-19 outbreak. Mixed teams composed of first year to senior residents from multiple subspecialties were important in assuring proper patient care and resident comfort, confidence, and safety. Training of personnel, reasonable working hours, and proper supervision were keys in attaining resident satisfaction and the reduction of burnout.

Internal Medicine residents showed extreme resilience and willpower as they experienced the crisis and helped patients. Internists and Internal Medicine residents are now a large part of our medical staff and one of the aspects we are proudest of with regard to work during the pandemic.

It is unlikely that this will be the last pandemic that many of our residents will live through. Limited data from previous pandemics have demonstrated that balancing optimal patient care and trainee education is a challenge that should be addressed in residency programs (11). The post-pandemic scenario will most likely highlight the need to readapt the length and core skills of medical residency programs. Supervisors should take the time now to address this concern and create careful and individual plans for each resident.

The challenges brought on by the COVID-19 outbreak and their direct impact on medical residency programs have yet to be measured, but experiences in working directly with patients and in the organization of the human resources are major learnings that can be used by the current generation of physicians in the next health crisis.

## APPENDIX

**Table:** Supplementary File

Team	Unit	Number of Beds	Specialties	Number of Residents
UTI - 11GN	ICU	12	Internal Medicine	10
			Critical Care	2
			Cardiology	1
			Endocrinology	1
UTI - 11GS	ICU	12	Internal Medicine	10
			Critical Care	2
UTI - 11FF	ICU	13	Infectious Disease	5
			Internal Medicine	5
			Critical Care	1
			General Surgery	1
UTI - 11EE	ICU	14	Internal Medicine	10
			Endocrinology	1
			Critical Care	1
			Pulmonology	6
UTI - 11DN	ICU	12	Internal Medicine	4
			Anesthesiology	4
			Rheumatology	2
			Plastic Surgery	2
			General Surgery	2
			Critical Care	1
UTI - 11DS	ICU	12	Internal Medicine	9
			Anesthesiology	4
			Psychiatry	1
			Critical Care	1
UTI - 04GN	ICU	14	OMFS	4
			General Surgery	5
			Geriatrics	2
			Plastic Surgery	1
UTI - 04DN	ICU	10	Abdominal Surgery	3
			General Surgery	2
			Neurology	2
			Endocrinology	1
UTI - 04GS	ICU	17	General Surgery	9
			Critical Care	3
			Rheumatology	2
			Plastic Surgery	2
UTI - 05DN	ICU	12	General Surgery	6
			Cardiology	4
			Emergency Medicine	2
			Critical Care	2
UTI - 05GS	ICU	10	Cardiology	8
UTI - 08AA	ICU	10	Internal Medicine	5
			Critical Care	3
UTI - 09DN	ICU	12	Internal Medicine	10
UTI - 09AA	ICU	16	Internal Medicine	9
			Gastroenterology	3
			Critical Care	2
UAC	ICU	22	Anesthesiology	12
			Thoracic Surgery	3
			Pediatrics	8
UTI - C4	ICU	16	Anesthesiology	4
			Geriatrics	6
			Neurology	2
			General Surgery	1
			OMFS	1
UTI - C3	ICU	20	Anesthesiology	8
			Geriatrics	3
			General Surgery	4
			Neurology	1
UTI - C1	ICU	20	Cardiology	3
			Oncology	4
			Pediatrics	6
			Psychiatry	1
			Rheumatology	1
			Genetics	1
UTI - C2	ICU	12	Anesthesiology	6
			Cardiology	2
			Neurology	1
			Psychiatry	1
03DN	Ward	26	Infectious Diseases	4
			Dermatology	4
			Ophthalmology	2
			Orthopedics	2
			Pediatrics	3
03DS	Ward	20	Infectious Diseases	4
			Dermatology	2
			General Surgery	2
			Pediatrics	1
			Orthopedics	3
04DS	Ward	20	Infectious Diseases	5
			General Surgery	2
			Orthopedics	2
			Dermatology	3
			Pediatrics	1
05DS	Ward	26	Palliative Care	6
			Acupuncture	2
			Forensic Pathology	2
06AA	Ward	24	Allergy & Immunology	3
			Pediatrics	2
			Dermatology	2
			Forensic Pathology	2
			Abdominal Surgery	3
			Orthopedics	2
06DS	Ward	25	Internal Medicine	2
			Gastroenterology	5
			Nuclear Medicine	1
			Pediatrics	2
			Psychiatry	2
			Sports Medicine	3
07AA	Ward	43	Internal Medicine	5
			Endocrinology	4
			Psychiatry	3
			Pediatrics	6
			Forensic Pathology	2
			Orthopedics	2
			Head and Neck Surgery	1
			Sports Medicine	1
07DS	Ward	27	Endocrinology	3
			Nuclear medicine	1
			Neurology	3
			Orthopedics	2
			Radiation Oncology	3
			Nuclear Medicine	1
07GN	Ward	25	Sports Medicine	3
			Obstetrics & Gynecology	2
			Rheumatology	3
			Urology	7
08DN	Onco-hematology Ward	24	Geriatric Medicine	2
			Hematology	6
			Allergy & Immunology	1
			Oncology	3
			Radiology	2
			Neurology	2
08DS	Ward	16	Orthopedics	3
			Pathology	1
			Pediatrics	4
			Radiology	2
			Rheumatology	2
08GS	Ward	30	General Surgery	3
			Endocrinology	1
			Geriatric Medicine	1
			Sports Medicine	2
			Rheumatology	2
			Orthopedics	2
			Pediatrics	1
			Nuclear Medicine	1
			Otorhinolaryngology	2
09GN	Ward	25	Infectious Diseases	5
			Dermatology	2
			General Surgery	3
09GS	Ward	28	General Surgery	2
			Geriatric Medicine	2
			Sports Medicine	2
			Nuclear Medicine	1
			Orthopedics	2
			Gynecology and Obstetrics	3
			Psychiatry	1
			Pathology	2
			Pediatrics	2
Obstetrics	Ward	20	Gynecology and Obstetrics	16
Pediatrics	Ward	5	Pediatrics	5
Psychiatry	Ward	8	Psychiatry	5
Total				227

OMFS: Oral and maxillofacial surgery.
